# Dental anomalies: prevalence and associations between them in a large sample of non-orthodontic subjects, a cross-sectional study

**DOI:** 10.1186/s12903-017-0352-y

**Published:** 2017-03-11

**Authors:** G Laganà, N Venza, A Borzabadi-Farahani, F Fabi, C Danesi, P Cozza

**Affiliations:** 1 Department of Orthodontics, Department of Clinical Sciences and Translational Medicine, University of Rome Tor Vergata, Via Montpellier, 1, 00133 Rome, Italy; 2 Private Practice of Orthodontics, London, England, UK; 3 Warwick Medical School, University of Warwick, Coventry, England, UK; 4 Private Practice, Rome, Italy

**Keywords:** Tooth anomalies, Non-orthodontic subjects, Panoramic radiograph

## Abstract

**Background:**

To analyze the prevalence and associations between dental anomalies detectable on panoramic radiographs in a sample of non-orthodontic growing subjects.

**Methods:**

For this cross-sectional study, digital panoramic radiographs of 5005 subjects were initially screened from a single radiographic center in Rome. Inclusion criteria were: subjects who were aged 8–12 years, Caucasian, and had good diagnostic quality radiographs. Syndromic subjects, those with craniofacial malformation, or orthodontic patients were excluded and this led to a sample of 4706 subjects [mean (SD) age = 9.6 (1.2) years, 2366 males and 2340 females]. Sample was subsequently divided into four subgroups (8, 9, 10, and 11–12 year-old groups). Two operators examined panoramic radiographs to observe the presence of common dental anomalies. The prevalence and associations between dental anomalies were also investigated.

**Results:**

The overall prevalence of dental anomalies was 20.9%. Approximately, 17.9% showed only one anomaly, 2.7% two anomalies, while only 0.3% had more than two anomalies. The most frequent anomalies were the displacement of maxillary canine (7.5%), hypodontia (7.1%), impacted teeth (3.9%), tooth ankylosis (2.8%), and tooth transposition (1.4%). The lower right second premolar was the most frequent missing teeth; 3.7% had only one tooth agenesis, and 0.08% had six or more missing tooth (Oligodontia). Mesiodens was the most common type of supernumerary tooth (0.66%). Two subjects had taurodontic tooth (0.04%). Tooth transpositions and displacement of maxillary canine were seen in 1.4 and 7.5%, retrospectively (approximately 69 and 58% were in the 8 and 9 year-old groups, retrospectively). Significant associations were detected between the different dental anomalies (*P* < .05).

**Conclusions:**

The results of our study revealed significant associations among different dental anomalies and provide further evidences to support common etiological factors.

**Electronic supplementary material:**

The online version of this article (doi:10.1186/s12903-017-0352-y) contains supplementary material, which is available to authorized users.

## Background

Dental anomalies caused by complex interactions between genetic, epigenetic and environmental factors during the process of dental development. This process is multifactorial, multilevel, and multidimensional [1]. Developmental dental anomalies may be caused by genetic and environmental factors in particular during the morpho-differentiation or histo-differentiation stages of tooth development [2].

The associations between dental anomalies have been a focus of a number of biologically enlightened clinical orthodontists. Many authors have contributed recently to our increased awareness of fundamental significant relationships among dental abnormalities [3].

Dental anomalies’ incidence and degree of expression in different population groups can provide important information for phylogenic and genetic studies, allowing us to understand variations within and between the different populations [4].

Understanding the process of morphogenesis and the variations in the outcomes is an important contribution to the multidisciplinary clinical team approach to treatment [5].

Early diagnosis allows optimal patient management and treatment planning and can reduce complications and the amount and complexity of the planned treatment.

If such associations of hereditary origin occur, they may be worth recognizing and studying, as early diagnosis of one tooth developmental disturbance may reveal a potential risk of future position or other teeth eruption disturbances. In fact, various dental anomalies of the dentition are frequently observed together in clinical practice [4].

Changes in the pattern of tooth eruption can affect the organization of the dental arches contributing to a malocclusion [6].

From a clinical point of view, the genetic component of the causative observed tooth disturbance can be investigated by evaluating the associated dental anomalies.

The aim of this study was to analyze the prevalence and the associations among dental anomalies detectable by panoramic radiographs in a relatively large sample of non-orthodontic growing subjects.

## Methods

For this cross-sectional investigation, digital panoramic radiographs of 5005 subjects were initially randomly selected from January 2006 to July 2015 from a single radiographic center in Rome. The radiographs were evaluated in the Department of Orthodontics at “Tor Vergata” University of Rome and after applying the exclusion criteria this led to a final sample of 4706 subjects with mean age of 9.6 (SD = 1.2) years [2366 males (M) and 2340 females (F)]. The study project was approved by the Ethic Committee at the University of Rome Tor Vergata and written consent was obtained from all subjects’ parents. The inclusion criteria were: subjects of 8 to 12 years of age, Caucasian, and subjects with good quality radiographs. In case of subjects with more than one panoramic radiographs, only the first one was evaluated. Exclusion criteria were: incomplete records (x-rays, clinical notes), syndromic and craniofacial subjects (e.g., cleft lip/palate), or a history of previous orthodontic treatment.

The final sample was divided into four subgroups as follows:

- The 8 year–old group: 1832 subjects (912 M and 920 F, mean age = 8.38 (0.28) years)
- The 9 year–old group: 1132 subjects (572 M and 560 F, mean age = 9.48 (0.28) years)
- The 10 year–old group: 890 subjects (450 M and 440 F, mean age = 10.48 (0.28) years)
- The 11 and 12 year–old group: 814 subjects (413 M and 401 F, mean age = 11.51 (0.28) years).

### Dental anomalies assessed and recorded

The presence of eight different anomalies was evaluated following these criteria:

- **Tooth agenesis or hypodontia (H)**: where no sign of crown calcification on the radiograph was evident and no evidence of loss attributable to caries, periodontal disease, or trauma could be seen. If missing teeth were suspected, we contacted the referring dentist to ascertain that subjects did not have history of extractions, syndromes, or craniofacial malformations. The lower limit of the age (8 years) in the present sample was chosen to study hypodontia of all permanent teeth, excluding third molars, with minimal false-positive findings [7].
- **Supernumerary tooth (ST)**: this was diagnosed when teeth were present in addition to the normal dentition [8].
- **Impacted tooth (IT)**: disturbance of eruption determinate by some physical barrier in their path local factors (lack of space, cysts or benign tumors, odontomas, persistent primary teeth) [9].
- **Tooth ankylosis (TA)**: a clinical condition whereby, after eruption, a tooth loses its ability to maintain the continuous eruptive potential as the jaws grow [10]. Radiographically and clinically evidenced by the presence of the infraocclusion.
- **Odontomas (O)**: a radiopaque mass which is a dental hamartoma composed of normal dental tissue that has grown in an irregular way. It presents in the compound form, by many little tooth-like structures held together, or, in the complex form, by a single amorphous mass [11].
- **Taurodontism (T)**: where the tooth body and/or pulp chamber enlarged vertically and pulp chamber is in a rectangular configuration [12].
- **Tooth transposition (TT)**: positional interchange of two adjacent teeth, or the development or eruption of a tooth in a position normally occupied by a non-adjacent tooth [13].
- **Displacement of maxillary canine (DMC)**: a condition wherein a maxillary canine does not follow its normal eruption path with asymmetry between the right and left maxillary canines; maxillary lateral incisor is late erupting, with evidence of resorption or proclination. Radiographically evaluated by the positional relationship between the maxillary canine cusp tip and adjacent lateral incisor and measurement of the angle formed by the long axis of the maxillary canine and the midline or the distance between the maxillary canine cusp tip and occlusal plane [14].

Digital panoramic radiographs were acquired with the same radiographic equipment (Orthophos XG; Sirona Dental Systems, Long Island City, NY using the following parameters: 65–90 kV, 15 mA, 13 s, 110 mGy cm, effective dose = 21.4 mSv). Images were stored in a digital database. Density and contrast enhancement tools adjusted these digital radiographs.

Images were evaluated independently by two different operators (N.V. and G.L.) on the computer monitor with subdued ambient lighting. To estimate the reproducibility of diagnosis, 100 radiographs selected randomly were examined once again separately by the two operators. The agreement between the operators was substantial (Kappa > 90).

### Statistical analysis

All descriptive and comparative statistical analyses were performed using the SPSS software package (Statistical Package for Social Sciences, version 16.0, SPSS Inc., Chicago, USA). The Spearman rank correlation coefficient was used to evaluate significant associations between the different dental anomalies. Any *P* value < .05 was considered as significant. The prevalence and the patterns of association were assessed among different dental anomalies. The findings of the significant associations between investigated dental anomalies and different abnormal teeth were further analyzed by the chi-square test. The Cohen’s kappa statistic was used between the two assessors to test the reproducibility of diagnosis. Descriptive statistics and frequency tables were then created for general descriptions of the results in the groups.

## Results

**Fig. 1:**
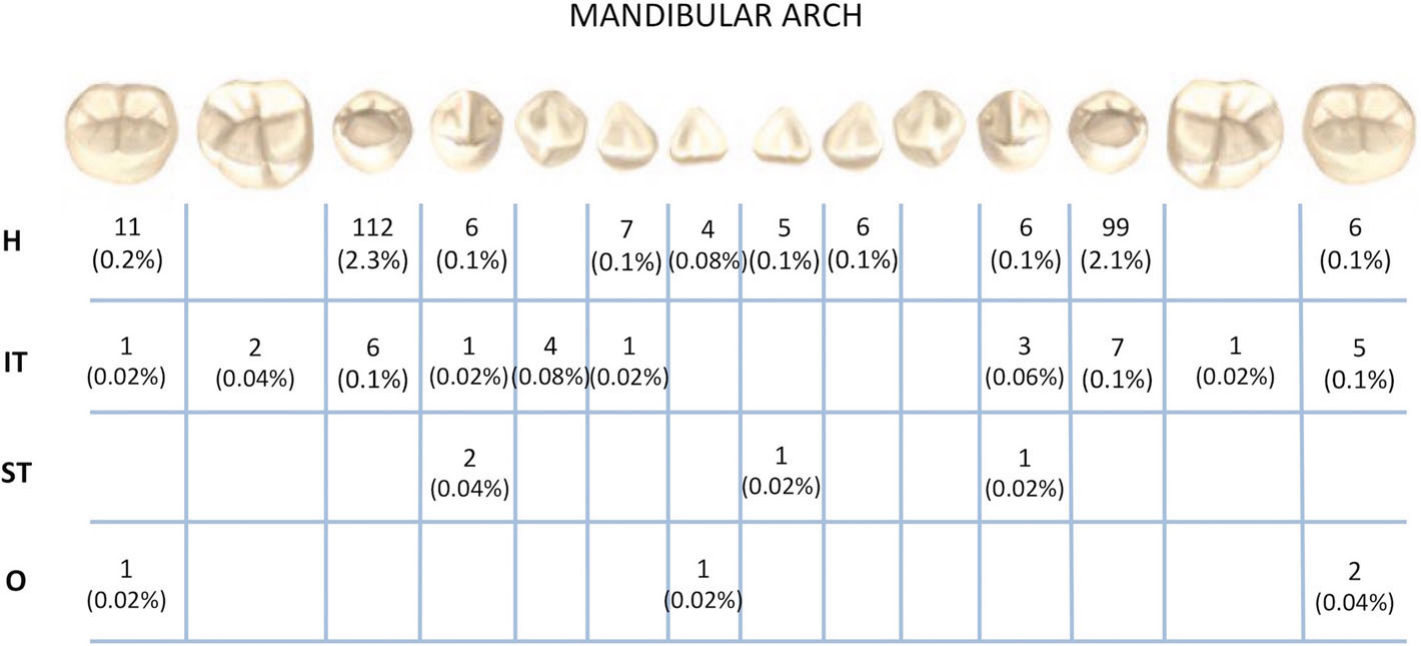
Number and prevalence of abnormal teeth found in maxillary arch. DMC: Displacement of maxillary canines; H: Hypodontia; IT: Impacted Teeth; TA: Tooth Ankylosis; TT: Tooth Transposition; ST: Supernumerary Teeth; (m): Mesiodens; O: Odontomas; T: Taurodontism

**Fig. 2:**
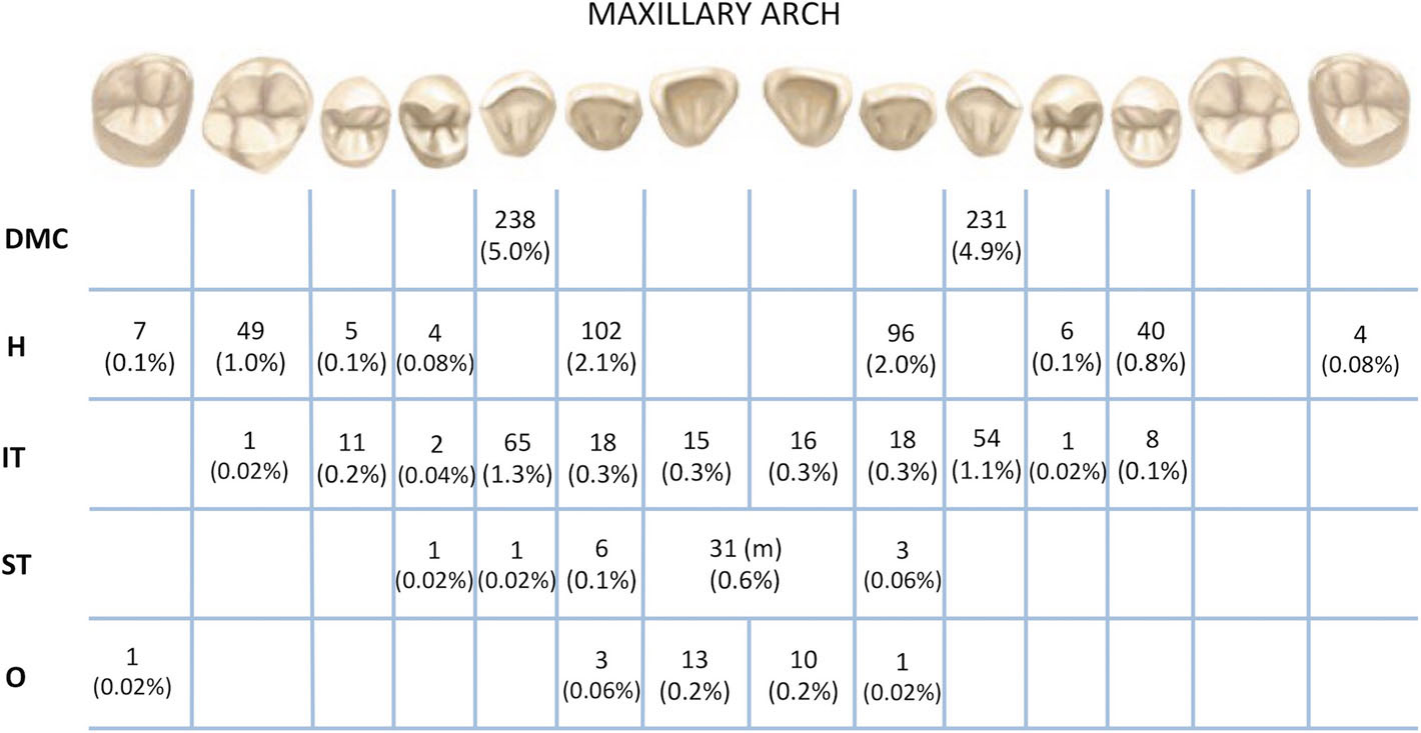
Number and prevalence of abnormal teeth found in mandibular arch. H: Hypodontia; IT: Impacted Teeth; ST: Supernumerary Teeth; O: Odontomas; T: Taurodontism

The prevalence rate of the different anomalies in the final sample is shown in the Table 1. Figures 1 and 2 show the distribution of different anomalies in the maxillary and mandibular arches. Cohen’s kappa statistic demonstrated substantial intra-examiner agreement between the two observers (Kappa > 0.90); no significant errors were found between the two analyses. The most frequent anomalies in the sample were the displacement of maxillary canine (7.5%), hypodontia (7.1%), impacted tooth (3.9%) and tooth ankylosis (2.8%). The overall prevalence of dental anomalies in the present sample was 20.9% (*n* = 984), with a male/female ratio of 1:1. Approximately, 17.9% (*n* = 842) of subjects showed only one anomaly, 2.7% (*n* = 126) two anomalies, while only the 0.3% (*n* = 16) more than two anomalies.

**Table 1:** Prevalence rate of different anomalies in the sample and in four sub-groups

	Whole SAMPLE		8 year–old group		9 year–old group		10 year–old group		11–12 year–old group	
	Prevalence n. (%)	M	Prevalence n. (%)	M	Prevalence n. (%)	M	Prevalence n. (%)	M	Prevalence n. (%)	M
		F		F		F		F		F
DMC	352 (7.5%)	M = 173	116 (6.3%)	M = 62	89 (7.8%)	M = 46	79 (8.8%)	M = 35	68 (8.3%)	M = 30
		F = 179		F = 54		F = 43		F = 44		F = 38
H	335 (7.1%)	M = 168	118 (6.4%)	M = 59	96 (8.4%)	M = 51	54 (6,0%)	M = 28	67 (8.2%)	M = 30
		F = 167		F = 59		F = 45		F = 26		F = 37
IT	185 (3.9%)	M = 87	72 (3.9%)	M = 35	40 (3.5%)	M = 19	39 (4.4%)	M = 17	34 (4.2%)	M = 16
		F = 98		F = 37		F = 21		F = 22		F = 18
TA	131 (2.8%)	M = 79	49 (2.6%)	M = 32	31 (2.7%)	M = 19	24 (2.7%)	M = 12	27 (3.3%)	M = 16
		F = 52		F = 17		F = 12		F = 12		F = 11
TT	67 (1.4%)	M = 39	34 (1.8%)	M = 19	12 (1.1%)	M = 7	8 (0.9%)	M = 4	13 (1.6%)	M = 9
		F = 28		F = 15		F = 5		F = 4		F = 4
ST	43 (0.9%)	M = 25	13 (0.7%)	M = 6	13 (1.1%)	M = 8	8 (0.9%)	M = 4	9 (1.1%)	M = 7
		F = 18		F = 7		F = 5		F = 4		F = 2
O	30 (0.6%)	M = 13	13 (0.7%)	M = 7	7 (0.6%)	M = 2	5 (0.6%)	M = 2	5 (0.6%)	M = 2
		F = 17		F = 6		F = 5		F = 3		F = 3
T	2 (0.04%)	M = 1	1 (0.1%)	M = 1	0	M = 0	0	M = 0	1 (0.1%)	M = 0
		F = 1		F = 0		F = 0		F = 0		F = 1

*DMC* Displacement of maxillary canines, *H* Hypodontia, *IT* Impacted Teeth, *TA* Tooth Ankylosis, *TT* Tooth Transposition, *ST* Supernumerary Teeth, *O* Odontomas, *T* Taurodontism

The lower right second premolar was the most frequent missing tooth. We detected 172 subjects (3.7%) with only one agenesis, 120 (2.5%) with bilateral agenesis, 39 (0.9%) with 3–5 agenesis, and 4 (0.08%) subjects with more than six missing teeth (Oligodontia).

A supernumerary tooth (ST) was found in 43 subjects (25 M, 18 F) (0.9%). Only 3 subjects had two supernumerary teeth (Table 2). Mesiodens was the most frequent type of supernumerary tooth (*n* = 31; 0.66%). The groups showing the highest number of this anomaly were the 9 and 11–12 year-old groups.

**Table 2:** Number of abnormal teeth in the same subject for each anomalies

	One abnormal tooth n. (%)	Two abnormal teeth n. (%)	Trhee or more abnormal teeth n. (%)
DMC	234 (5,0%)	118 (2.5%)	0
H	172 (3.7%)	120 (2.5%)	43 (0.9%)
IT	133 (2.8%)	45 (1,0%)	7 (0.1%)
TA	60 (1.3%)	50 (1.1%)	21 (0.4%)
TT	57 (1.2%)	10 (0.2%)	0
ST	40 (0.8%)	3 (0.1%)	0
O	29 (0.6%)	0	1 (0.0%)
T	2 (0.0%)	0	0

*DMC* Displacement of maxillary canines, *H* Hypodontia, *IT* Impacted Teeth, *TA* Tooth Ankylosis, *TT* Tooth Transposition, *ST* Supernumerary Teeth, *O* Odontomas, *T* Taurodontism

An impacted tooth (IT) was detected in 185 subjects (3.9%) and none of the impacted tooth caused by cyst or benign tumors. Maxillary impaction was found in 161 subjects, whereas only 24 subjects showed mandibular impacted tooth. The most commonly impacted teeth were: maxillary canines (*n* = 119; 2.5%), maxillary lateral incisors (*n* = 34; 0.7%), and maxillary central incisors (*n* = 33; 0.7%). The most frequent ankylosed teeth were the lower second deciduous molars (*n* = 109; 2.3%), lower first deciduous molars (*n* = 80; 1.7%) and the upper second deciduous molars (*n* = 27; 0.6%). Odontomas (O) were found mainly in the maxillary anterior region (27 of 32 cases in the central incisor region). We detected two subjects with a taurodontic tooth (0.04%) and no significant correlations with other anomalies were found.

The unilateral displacement of maxillary canine (DMC) was identified in 234 subjects (5.00%), while bilateral DMC in 118 subjects (2.50%). DMC subjects were constantly found in all groups except for the 8-year–old group, with the lower prevalence (6.23%).

Overall, we detected 67 (1.4%) cases with transpositions. The maxillary canine-first premolar transposition was the most frequent type (*n* = 44; 0.93%). We also detected 25 maxillary first premolar-second premolar transpositions (0.53%) and four maxillary canine-lateral incisor transpositions (0.08%). The M/F ratio in the subjects with transposition was 39:28. Only ten cases with bilateral transposition were observed. There were significant associations among different anomalies (*P* < .01, Table 3). Table 4 shows significant associations among different abnormal teeth.

**Table 3:** Associations among different anomalies and the corresponding *p* values, correlation coefficients and 95% confidence interval

	ST	O	H	IT	DMC	TA	TT
ST	–	.162 .020 -.045–.086	.080 .026 -.040–.091	*P* < *0.01* *.038* -.028–.103	.479 -.010 -.076–.055	.854 -.003 -.068–.063	*P* < *0.001* *.* *083* .017–.148
O		–	.923 -.001 -.067–.064	*P* < *0.001* *.162* .097–.228	.387 -.013 -.078–.053	.353 -.014 -.079–.052	.376 0.13 -.053–.078
H			–	.409 .012 -.053–.077	*P* < *0.01* *.044* -.022–.109	.051 .029 -.037–.094	*P* <* 0.001* *.057* .009–.122
IT				–	.740 .005 -.061–.070	*P* <* 0.05* *.132* -.034–.097	*P* < *0.001* *.068* .002–.133
DMC					–	*P* < *0.01* *.040* -.026–.105	.996 .000 -.066–.065
TA						–	.110 .023 -.042–.089
TT							–

Spearman rank correlation coefficient. *P* value < .05 was considered as significant

*DMC* Displacement of maxillary canines, *H* Hypodontia, *IT* Impacted Teeth, *TA* Tooth Ankylosis, *TT* Tooth Transposition, *ST* Supernumerary Teeth, *O* Odontomas, *T* Taurodontism

**Table 4:** Significant associations among different abnormal teeth

Abnormal tooth	Significant associations (*P* < .01)
Hypodontia 12	Hypodontia 15, 22, 25, 35, 45; DMC 13, 23
Hypodontia 15	Hypodontia 12, 22, 25, 35, 45; Ankylosis 85
Hypodontia 22	Hypodontia 12, 15, 25, 35, 45; DMC 13, 23
Hypodontia 25	Hypodontia 12, 15, 22, 35, 45
Hypodontia 35	Hypodontia 12, 15, 25, 22, 45
Hypodontia 45	Hypodontia 12, 15, 22, 25, 35
Impacted 13	Impacted 23
Impacted 23	Impacted 13
DMC 13	Hypodontia 12, 22; DMC 23; Ankylosis 75
DMC 23	Hypodontia 12, 22; DMC 13
Ankylosis 74	Ankylosis 75, 84, 85
Ankylosis 75	DMC 13; Ankylosis 74, 84, 85
Ankylosis 84	Ankylosis 74, 75, 85
Ankylosis 85	Hypodontia 15; Ankylosis 74, 75, 84

Spearman rank correlation coefficient

## Discussion

The present study analyzed the prevalence and the pattern of associations of different dental anomalies in a large sample of non-orthodontic subjects. Numerous studies evaluated the prevalence of dental anomalies in orthodontic or paediatric subjects. The nature of the examined subjects influenced prevalence rates of the examined anomalies, but it did not necessarily reflect the prevalence in the general population. The present study design was such that overcame some methodological drawbacks of previous investigations on associated dental anomalies. Furthermore, no studies in Italy analyzed dental anomalies on such a high number of non-orthodontic subjects.

The aim of the present investigation was to provide further evidence on reciprocal associations and distribution of different dental anomalies in a large population of growing subjects.

The findings of the present study revealed significant associations among different dental anomalies and this may support a common etiological origin for these conditions.

Esenlik et al. [15], in a study of a Turkish population, analyzed 2599 radiographs of subjects with a similar age range. They found a prevalence rate of ST of 2.7%. The literature analysis suggests a prevalence rate of ST to be between 0.2 and 3% [16]. These findings are similar to ours showing a prevalence rate of 0.9%. Furthermore, we found that the 8 year-old group had the lowest prevalence of ST. This result could be considered as a false positive data, related to a delayed ST development [8]. Present study revealed significantly higher prevalence of ST in the maxillary arch (0.89%), than in the mandibular arch (0.01%). The most common dental anomaly associated with ST was the IT and TT, similar to authors who described associations with the displacement of a permanent tooth and failure of eruption [5, 17]. The higher prevalence of tooth impactions in the present sample could be partially explained by a higher proportion of these cases detected in the 8 and 9 year-old groups (~60%) and it is possible that some of these cases normalize later on and do not present as the tooth impactions.

In a review of Rakhshan [18], he estimated the prevalence of agenesis in permanent dentition, excluding third molars, to be in a range of 0.15 and 16.2%. Similarly, authors revealed IT, DMC and T as the most frequent associated dental anomalies [18]. The prevalence of H in the present sample was 7.1%, which is well within the same range [18] and showed significant associations with the DMC and TT. As described in the previous article we demonstrated that the missing elements are often the distal teeth in each group of homogeneous teeth: upper and lower third molars, lateral incisors and lower second premolars [19]. In particular, agenesis of maxillary lateral incisors was significantly associated with DMC. The present findings contrast with Peck’s findings reporting that agenesis of the mandibular second premolars is more prevalent than agenesis of the maxillary lateral incisors [20]. Moreover, contradictory to Al-Abdallah’s conclusions, we demonstrated that mandibular hypodontia was not significantly associated with IT and TA [7].

Very few studies described the prevalence of taurodontism: the most recent one was conducted in a Trinidad and Tobago’s population and revealed an incidence of 4.79%. This is significantly higher than ours (0.04%) and it could be due to racial difference and differences in diagnostic methods [21].

A considerably higher prevalence of tooth transpositions in the present general population (1.4%) was found, compared with Papadopulos’ meta-analysis (0.33%) [22]. Moreover, present findings suggest a higher prevalence in male subjects. Peck and Peck [23] suggest that transposition equally affects both sexes, while others, similar to our findings, have found a higher prevalence in males [24]. The higher prevalence of tooth transpositions could be partially explained by a higher proportion of these cases detected in the 8 and 9 year-old groups (~69%) and it was possible that some of these cases normalized later on and did not present the tooth transpositions. Significant associations reported in the literature were among agenesis, microdontic teeth and tooth ankylosis. The correlation test showed a significantly higher percentage of ST (*P* < .001), H (*P* < .001) and IT (*P* < .001).

Prevalence of deciduous molar ankylosis has been reported in previous studies with some variability; to be between 1.3 and 38.5% [25]. This variability is probably due to different inclusion criteria, age of the sample, and racial differences between studied sample populations. Our study identified significant associations between ankylosis of mandibular deciduous molars and displacement of maxillary canines (*P* < .01).

The prevalence of displaced maxillary canines in the Caucasian population is reported to be about 2–3% [26]. In this non-orthodontic sample, the prevalence was 7.5%. Male-to-female ratio of DMC in the present study was 1:1 and bilateral-to-unilateral ratio was 1:2, with a bilateral occurrence of 2.50%. By contrast, Peck et al. [27] reported that DMC occurred twice in females than in males and the bilateral occurrence was reported to be in the range of 19–45%. Similar to tooth transposition, the higher prevalence of displaced maxillary canines could be due to a higher proportion of these cases being detected in the 8 and 9 year-old group (~58%) and it is possible that some of these cases normalized later on and did not present as displaced maxillary canines.

## Conclusions

- Numerous and significant associations between different dental anomalies were found. In particular, significant associations were detected between Supernumerary teeth and Impacted teeth, Tooth transposition; Odontomas and Impacted teeth; Hypodontia and Displacement of maxillary canines, Tooth Transposition; Impacted Teeth and Tooth Ankylosis, Tooth Transposition; Displacement of maxillary canines and Tooth Ankylosis. These results may suggest common etiological factors for these conditions.
- The present findings can be used in estimation of prevalence of common dental anomalies in the Italian population.

## Additional file

Additional file 1: Analyzed data. Anonymous data used for the study. (XLSX 317 kb)

## Abbreviations

(m): Mesiodens
CI: Confidence interval
DMC: Displacement of maxillary canines
H: Hypodontia
IT: Impacted teeth
O: Odontomas
SD: Standard deviation
ST: Supernumerary teeth
T: Taurodontism
TA: Tooth ankylosis
TT: Tooth transposition
